# Pregnancy has no significant impact on the prognosis of differentiated thyroid cancer

**DOI:** 10.20945/2359-3997000000413

**Published:** 2021-11-11

**Authors:** Gabriela Maia Nobre, Mariana Yoshii Tramontin, Natalia Treistman, Paulo Alonso Alves, Fernanda Accioly Andrade, Daniel Alves Bulzico, Rossana Corbo, Fernanda Vaisman

**Affiliations:** 1 Instituto Nacional do Câncer Serviço de Oncologia Endócrina Rio de Janeiro RJ Brasil Serviço de Oncologia Endócrina, Instituto Nacional do Câncer (INCA), Rio de Janeiro, RJ, Brasil; 2 Universidade Federal do Rio de Janeiro Faculdade de Medicina Programa de Pós-Graduação em Endocrinologia Rio de Janeiro RJ Brasil Programa de Pós-Graduação em Endocrinologia, Faculdade de Medicina, Universidade Federal do Rio de Janeiro (UFRJ), Rio de Janeiro, RJ, Brasil

**Keywords:** Pregnancy, thyroid cancer, response to therapy, progression

## Abstract

**Objective:**

To evaluate the impact of pregnancy on differentiated thyroid carcinomas (DTC) behavior

**Subjects and methods:**

Retrospective study of patients diagnosed with DTC before or during pregnancy and treated with standard therapy. In women diagnosed with DTC before pregnancy, we evaluated the occurrence of progression according to categories of response to therapy based on imaging and non-stimulated thyroglobulin (TG) levels.

**Results:**

Of 96 analyzed patients, 76 became pregnant after DTC treatment and 20 were diagnosed with DTC during pregnancy. Among women who became pregnant after a DTC diagnosis, no difference was observed regarding response to therapy before and after pregnancy. Disease progression after pregnancy was documented in six of these patients, while seven of them presented progression before pregnancy but were only treated after delivery. Patients with DTC diagnosed during pregnancy had a higher rate of distant metastases at diagnosis (30%) compared with the patients who became pregnant after DTC diagnosis (9.2%, p = 0.01).

**Conclusion:**

Pregnancy had no impact on the natural course of DTC. Disease progression after pregnancy was limited and probably related to more aggressive disease and higher risk stratification at diagnosis. Still, mild disease progression may have occurred asymptomatically in some patients.

## INTRODUCTION

Differentiated thyroid cancer (DTC) is very common in women of reproductive age and one of the most frequent cancers diagnosed during pregnancy. Pregnancy seems to stimulate the development of existing neoplastic thyroid cells, especially in women with a history of thyroid pathology or genetic predisposition (1). More than a third of all women diagnosed with thyroid cancer are of childbearing age; thus, the impact of pregnancy on DTC behavior is a pertinent issue (2). Physiological changes during pregnancy stimulate the enlargement of maternal thyroid volume and pre-existing thyroid nodules. Indeed, some pregnancy-associated factors are known to stimulate the thyroid (3). For example, both the hormones beta-human chorionic gonadotropin (β-hCG) and estrogen have a transient stimulatory effect on thyroid cells, which partially explains the thyroid enlargement observed during pregnancy (4,5). Pregnancy also stimulates the production of thyroglobulin (TG). In women without any thyroid pathology, TG values are higher in the third trimester of pregnancy compared with the first year postpartum (6,7).

Many physicians consider the diagnosis of thyroid cancer in women of childbearing age to be challenging, since decisions regarding surgery, radioiodine therapy, and surveillance must take into account the patient’s pregnancy plans. Only a few studies in the literature have addressed the treatment of DTC diagnosed during pregnancy and the surveillance of pregnant women with a history of DTC. Some authors report a higher frequency of diagnosis of benign and malignant tumors in thyroid nodules detected during pregnancy, with values of up to 80% for all neoplasms, 37%-63% for benign nodules, and 28.5%-43% for DTC (8,9). Cases of DTC detected during pregnancy usually comprise papillary carcinomas; these tumors affect 1 in 1,000 pregnant women and have a course similar to that in non-pregnant women (10). A few cases require surgical intervention during pregnancy, which should be performed preferably during the second trimester and by an experienced surgeon (10).

Pregnancy has also been associated with an increased risk of DTC recurrence or progression in women previously treated for this malignancy. However, the impact of pregnancy may differ depending on the DTC’s response to therapy before pregnancy (11). Surveillance guidelines by the 2015 American Thyroid Association (ATA) vary according to the response to therapy dynamic risk stratification, which modifies the initial risk of recurrence based on data accumulated during follow-up (12,13). Several studies have validated this dynamic risk stratification system (14-16), but only a few have done so in the context of pregnancy after complete DTC therapy (5,7). Previous studies have shown that pregnancy has no relevant impact on DTC recurrence in women without evidence of biochemical or structural disease before pregnancy (17-19). However, nonsignificant progression has been observed in women with structural or biochemical incomplete response before pregnancy and, even in these patients, progression is generally mild and requires no additional therapy in the first year postpartum (7,20). These data suggest that the response to therapy status before pregnancy may be an important predictor of disease progression during pregnancy. A retrospective study by Leboeuf and cols. (7) in 36 women showed no evidence of recurrence in the postpartum period in women without suspected images on ultrasound and undetectable or low TG levels prior to pregnancy. However, one out of three women with known suspected cervical images presented an enlargement of a previously stable local lymph node, and one woman without evidence of progression of structural disease and stable lung metastases showed an increase in TG level. Additionally, eight patients presented TG values 20% higher after delivery compared with pre-delivery levels (three had structural disease and five had no evidence of disease), which had no short-term clinical implication in the postpartum period.

Due to the small number of studies addressing the associations between pregnancy and DTC and the controversies highlighted above, the aim of this study was to evaluate the impact of pregnancy on DTC behavior in a Brazilian cohort of women diagnosed with DTC before or during pregnancy.

## SUBJECTS AND METHODS

The study project was approved by the institution’s research ethics committee with CAAE number 03487712.5.3001.5257.

This was a retrospective study of women diagnosed with DTC, who became pregnant after treatment with standard therapy (total or subtotal thyroidectomy — with or without neck dissection — and radioiodine therapy) or were diagnosed with DTC during pregnancy and treated after delivery. The patients’ records were obtained from the database of the *Instituto Nacional do Câncer* (INCA) between 1986 and 2020. We excluded patients diagnosed with DTC before the age of 18 years, those with anaplastic and medullary thyroid cancer, and women who had still not delivered before the study analysis. We only included those women who had a minimum follow-up of 1 year after delivery, to allow time to observe the impact of pregnancy on outcomes. In women with several pregnancies, we considered for the analysis the first pregnancy after DTC diagnosis and treatment.

The initial staging (TNM) was analyzed taking into account the initial tumor size and presence of lymph node and distance metastases at diagnosis according to the 2018 American Joint Committee on Cancer (AJCC, 8th edition), as well as the initial risk stratification of recurrence or persistence according to the 2015 ATA guidelines. We also investigated the tumor’s histological subtype, type of treatment received (total thyroidectomy with radioiodine [^131^I] therapy, total or subtotal thyroidectomy with or without neck dissection), and whether additional therapy was delivered within the first 18 months after delivery. Finally, we evaluated the occurrence of structural or biochemical disease progression after pregnancy and the final disease status at the last medical visit.

### Definitions of dynamic stratification based on response to therapy

Response to therapy was evaluated 1 year before and 1 year after pregnancy and was categorized as follows: excellent response (no evidence of structural or biochemical disease, undetectable anti-TG levels, and TG levels < 0.2 ng/mL on levothyroxine suppression), biochemical incomplete response (no evidence of structural disease and TG level > 1 ng/mL on levothyroxine suppression), structural incomplete response (locoregional persistence or distant metastases), or indeterminate response (TG level > 0.2 ng/mL or < 1 ng/mL on levothyroxine suppression, stable or declining antibody levels, and nonspecific findings on imaging methods). We considered for the analysis, in general, the last category established before pregnancy and the first one after pregnancy. Levels of thyrotropin (TSH), TG, and anti-TG antibodies available 1 year before and 1 year after pregnancy were also assessed, along with the occurrence of detectable structural disease on imaging 1 year before and 1 year after pregnancy. We also considered for the analysis the last laboratory tests and images obtained before pregnancy and the first ones obtained after pregnancy showing changes from baseline (before pregnancy). Any suspicious image on ultrasound, computed tomography (CT), or positron emission tomography (PET)-CT was considered to be a structural disease regardless of histological or cytological confirmation.

### Definitions of biochemical and structural progression

Based on the collected information described above, we evaluated the occurrence of structural or biochemical progression. In patients with structural incomplete response, progression was defined as an increasing size of a known lesion or identification of a new metastatic focus on imaging. Biochemical progression was considered present when patients on levothyroxine suppression presented increasing TG levels from a baseline of 1 ng/mL as follows: (A) if previous TG 1.1-10 ng/mL, or (B) > 20% increase if previous TG > 10 ng/mL, or (C) any increase in anti-TG values in patients with positive antibodies. In patients who underwent thyroid lobectomy as the surgical treatment, only structural progression was assessed.

In women with progressive disease, we calculated the tumor volume doubling time (DT) and the TG DT using the last four volume measurements before pregnancy and all the available measurements obtained within the first year after delivery. For the TG DT, the patients were required to have appropriate TSH suppression and negative anti-TG antibodies. We used the Kuma Hospital Calculator to estimate both the tumor volume and TG DT values (21).

Target TSH values during pregnancy were between 0.1-0.5 mIU/L for patients with biochemical or structural evidence of disease before pregnancy and 0.5-1.0 mIU/L for those without evidence of disease before pregnancy. For the analysis of biochemical progression, we only considered TG values when TSH levels were adequately suppressed, as recommended by the ATA guidelines (12).

Our institution used different TG assays during the study period. The functional sensitivity of the assay was ~1 μg/L between 1986 and 1997 and 0.5 μg/L between 1998 and 2001. From 2001 onwards, TG levels were measured using an immunometric assay (Immulite 1000 Immunoassay System; Siemens Healthcare Diagnostics Inc., Tarrytown, NY, USA) with a functional sensitivity of 0.2 μg/L between 2001 and 2014 and 0.1 μg/L after 2014.

Similarly, various anti-TG assays were used during the study period. The functional sensitivity of the assay was ~60 IU/L between 1986 and 2009 and 20 IU/L from 2009 to 2013. From 2014 onwards, serum anti-TG levels were measured using an electrochemiluminescence assay with a functional sensitivity of 10 IU/L.

### Statistical analysis

Normality was analyzed with the Kolmogorov-Smirnov test. Numeric variables with non-normal distribution are presented as median and range values and those with normal distribution as mean and standard deviation values. A simple exploratory analysis was conducted using a Cox model to identify the first-year and long-term outcomes. The variables emerging as significant in this analysis were evaluated in a multivariate model and are presented as hazard ratios (HRs) and 95% confidence intervals (CIs). Unadjusted and adjusted HRs were compared to verify differences in magnitudes that could indicate a modifying effect or confounders. Proportional hazards assumption was verified using Schoenfeld residuals. All analyses were performed using the software Statistical Package for the Social Sciences for Windows, version 20.0 (IBM Corporation, Armonk, NY, USA). P values below 0.05 indicated statistical significance.

## RESULTS

### Clinical features

**Figure 1:**
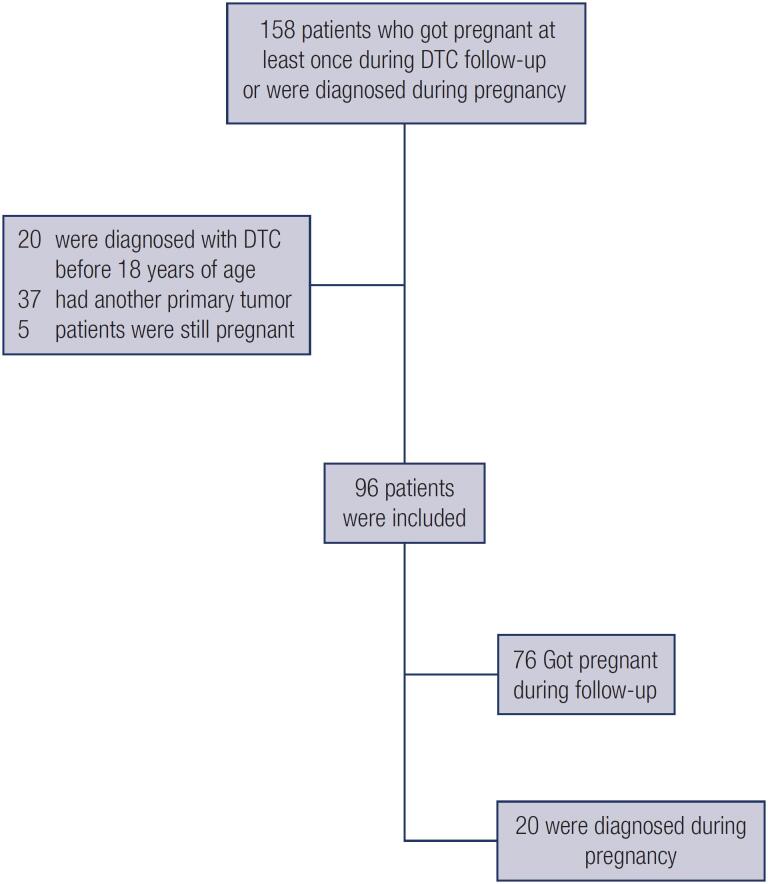
Flow diagram of the process of selection of the participants.

Between 1972 and 2019, we identified 158 women diagnosed with DTC before or during pregnancy. Of these, 20 were excluded due to a DTC diagnosis before the age of 18 years, 37 due to diagnoses of primary tumors in locations different than the thyroid, and five due to ongoing pregnancy at data collection. The final analysis included 96 women, of whom 76 were diagnosed with DTC before pregnancy and 20 received this diagnosis during pregnancy (Figure 1).

The overall cohort had a median age of 27 years (18-40 years) at diagnosis and 32 years (20-44 years) at pregnancy. The median follow-up time was 11 years (1-47 years) (Table 1). Among participants with DTC diagnosed before pregnancy, the median interval between diagnosis and delivery was 4.8 years (0.9-19 years). Eleven patients became pregnant more than once during follow-up (eight patients had two pregnancies and three patients had three pregnancies), yielding a total of 110 pregnancies, 90 of which occurred after a DTC diagnosis.

**Table 1:** Clinical presentation of DTC diagnosed during pregnancy vs before pregnancy

	DTC Diagnosed during pregnancy (N=20)	DTC Diagnosed before pregnancy (N=76)	Entire cohort(N=96)
Age at diagnosis (median-range)	31 years (20-40)	26 years (18-40)	27 years (18-40)
Age at pregnancy (median-range)	31 years (20-40)	32 years (21-44)	32 years (20-44)
Histology			
PTC	9 (45%)	58 (76%)	67 (69.8%)
FVPTC	9 (45%)	14 (18%)	23 (24%)
FTC	1 (5%)	2 (2.6%)	3 (3.1%)
Other	1 (5%)	2 (2.6%)	3 (3.1%)
Size of primary tumor (median-range)	2.5 cm (0.8-5.5)	2.2 cm (0.3-7.5)	2.2 cm (0.3-7.5)
Total thyroidectomy	20 (100%)	73 (96%)	93 (96.9%)
Lymph node metastases at diagnosis			
N0	10 (50%)	36 (47%)	46 (47.9%)
N1a	1 (5%)	12 (18%)	13 (13.5%)
N1b	9 (45%)	28 (37%)	37 (38.5%)
Distant metastases	6 (30%)	7 (9.2%)	7 (9.2%)
ATA recurrence risk			
Low	3 (15%)	20 (26.3%)	23 (24%)
Intermediate	8 (40%)	38 (50%)	38 (50%)
High	9 (45%)	18 (23.7%)	27 (28.1%)
Response to therapy at last visit			
Excellent	9 (45%)	36 (47%)	45 (46.9%)
Indeterminate	3 (15%)	16 (21%)	19 (19.8%)
Biochemical incomplete	2 (10%)	12 (16%)	14 (14.6%)
Structural incomplete	6 (30%)	12 (16%)	18 (18.8%)
Final Status at last visit			
No evidence of disease	9 (45%)	32 (42%)	41 (42.7%)
No evidence of disease after additional therapy	0	4 (5.2%)	4 (4.2%)
Stable disease	9 (45%)	34 (44.7%)	8 (8.3%)
Progressive disease	2 (10%)	6 (7.9%)	
Follow-up (median-range)	13 years (1-33)	11 years (1-47)	11 years (1-47)

Among women diagnosed with DTC during pregnancy, the median time between delivery and surgery was 6 months and 20 years. None of these patients underwent surgery during pregnancy, based on decisions by the medical team. These patients were asymptomatic regarding the disease and had slow growing nodules. The presence of distant metastases could not be assessed during pregnancy due to the risk of radiation exposure. One patient who was diagnosed with DTC during pregnancy declined treatment and was lost to follow-up. She returned to our institution 20 years later due to tumor growth, underwent surgery, and currently has no evidence of disease.

In the entire cohort, the initial treatment comprised subtotal (n = 3, 3.1%) or total (n = 93, 96.9%) thyroidectomy followed by radioiodine therapy (n = 71, 73.7%). Among the histological subtypes observed, papillary cancer was the most common (n = 67, 69.8%), followed by the follicular variant of papillary carcinoma (n = 23, 24%), follicular carcinoma (n = 3, 3.1%), and other subtypes (n = 3, 3.1%) including oxyphilic, insular, and tall-cell carcinoma (one case each). The tumors had an initial median size of 2.2 cm (0.3-7.5 cm). At diagnosis, 50 patients had local lymph node metastases (13.5% N1a and 38.5% N1b) and 13 patients (13.5%) had distant metastases, mainly to the lungs. The risk of recurrence at diagnosis (2015 ATA guidelines) was low in 24.0% (n = 23) of the patients, intermediate in 47.9% (n = 46), and high in 28.1% (n = 27) of them.

Among patients who became pregnant after the DTC diagnosis, 15 (19.7%) received additional therapy within 1 year after the delivery. Pregnancy complications were reported in 11 patients and included hypertensive disorders of pregnancy in six of them (Table 1).

On the last medical visit, 42.7% of the patients had no evidence of disease without further treatment, 4.2% had no evidence of disease after further treatment, 44.8% had stable disease, and 8.3% had progressive disease.

### Responses to therapy in patients diagnosed with DTC before versus during pregnancy

**Figure 2:**
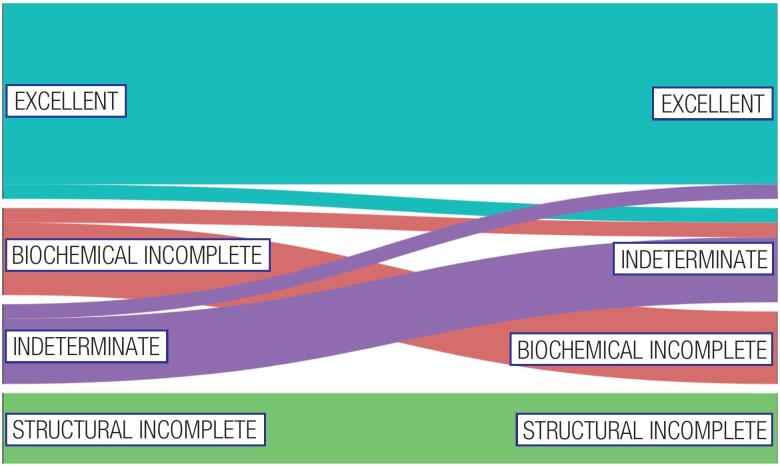
Response to therapy assessments before and after pregnancy (n = 76). Legend: Of the patients with an “excellent response” prior to pregnancy, two were reclassified as having an “indeterminate response”. Of patients with “indeterminate response” before pregnancy, three were reclassified as having an “excellent response”, and of those with biochemical incomplete response, two were reclassified to the “indeterminate response” category. All patients with structural disease remained in the same category after pregnancy.

Among patients who became pregnant after a DTC diagnosis, the responses to therapy prior to the pregnancy were as follows: excellent response, 39.5%; indeterminate response, 18.5%; biochemical incomplete response, 21.0%; and structural incomplete response, 21.0% (Table 2). The rates of each type of response to therapy were comparable between patients diagnosed with DTC before or during pregnancy. Of the patients with excellent response prior to the pregnancy, most maintained the same response, while two patients were reclassified as having an indeterminate response based on TG values and unspecific ultrasonographic findings. In contrast, among patients with indeterminate response before pregnancy, three were reclassified as having an excellent response, while the classification continued the same in the remaining patients. Among patients with biochemical incomplete response, most maintained the same category after pregnancy, while two patients were reclassified to the indeterminate response category. As expected, all patients with structural disease remained in the same category of response to treatment after pregnancy (Figure 2). No significant differences were observed in median TG and anti-TG values before and after pregnancy (Table 2).

**Table 2:** Impact of pregnancy on thyroid cancer outcomes (76 women diagnosed and treated before pregnancy)

	Before pregnancy	After pregnancy	p-value
Non stimulated Tg (ng/mL) (median-range)	0.2 (<0.04-262)	0.4 (<0.04-2141)	0.54
Anti-Tg (ng/mL) (median-range)	Undetectable (undect.-3997)	Undetectable (undect.->4000)	0.066
Response to therapy			
Excellent	30 (39.5%)	31 (40.8%)	1.0
Indeterminate	14 (18.5%)	15 (19.7%)	1.0
Biochemical incomplete	16 (21%)	14 (18.5%)	0.83
Structural incomplete	16 (21%)	16 (21%)	1.0
Disease Status after 1 year of pregnancy			
No evidence of disease	30 (39.5%)	31 (40.8%)	1.0
Stable disease	39 (51.3%)	32 (42.1%)	0.32
Progressive disease	7 (9.2%)	13 (17.1%)	0.22

Tg: thyroglobulin.

Anti-Tg: thyroglobulin antibody.

### Assessment of the impact of the pregnancy on DTC

Thirteen patients (17.1%) diagnosed with DTC before pregnancy were treated for disease progression after pregnancy; seven of them presented biochemical progression and six presented structural progression (Table 3). Most of these patients had mild and asymptomatic progression related to a slight increase in tumor volume. Seven patients in this group had disease progression that was documented and treated (Table 2). The tumor volume DT and the TG DT presented no significant changes after pregnancy compared with values before pregnancy.

**Table 3:** Progressive patients

	1	2	3	4	5**	6**	7	8	9	10	11	12	13
Ageat diagnosis (years)	19	24	24	27	28	29	31	32	26	27	26	20	19
Age atpregnancy (years)	23	25	25	32	30	35	39	38	29	32	30	25	21
SizeT(cm)	2.4	4.5	3	2	3	1.7	4.4	2	2.5	1.5	Unknown.	Unknown.	2.5
Nstatus	Yes	No	Yes	Yes	Yes	Yes	Yes	Yes	Yes	No	Yes	Yes	Yes
Mstatus	No	Yes(Lung)	No	Yes(Lung)	No	No	Yes(Lung)	No	No	No	No	Yes	No
ATA2015	High	High	Intermed.	High	Intermed.	Intermed.	High	High	High	Low	High	High	High
Response to initial therapy before pregnancy	Structural incomplete	Structural incomplete	Structural incomplete	Structural incomplete	Biochem incomplete	Indeterm.	Structural incomplete	Biochem. incomplete	Indeterm.	Biochem. incomplete	Structural incomplete	Structural incomplete	Structural incomplete
Place of progression	Local	Lung	Local (>50%)	Lung	Biochem.	Biochem	Lung	Biochem	Biochem	Biochem	Local (>50%)	Biochem	Biochem
Additional Therapy	Neck dissection + RAI	RAI	Neck dissection + RIT	Observation	RAI	Observation	Observation	Observation	Observation	Observation	Neck dissection	RAI	RAI
Non stimulated Tg before pregnancy (ng/ml)	Unknown.	Unknown.	41	2,4	0.6	0.2	0.1	9.7	3.76	1.49	2.94	2.5	3.8
Anti Tg before pregnancy (ng/ml)	Unknown.	Unknown.	Negative	Negative	90	112	3997	Negative	Negative	Negative	Negative	Unknown	Negative
Initial size of metastatic site	0.5	1.0	1.1	0.7	N/A	N/A	0.4	N/A	N/A	N/A	1.7	0.5	0.4
Tumor volume DT before pregnancy	12.6	10.8	4.5	33.8	N/A	N/A	7.3	N/A#	N/A#	N/A#	7.8	infinite	infinite
Tumor volume DT after delivery	5.7	11.9	6.7	45.7	N/A	N/A	8.2	N/A#	N/A#	N/A#	8.1	infinite	infinite
Tg DT before pregnancy	N/A	N/A	6.9	12.5	N/A*	N/A*	N/A*	6.4	10.8	12.7	4.6	62.3	102.8
Tg DT after delivery	2.4	3.1	5.6	14.6	N/A*	N/A*	N/A*	7.2	9.8	13.3	5.7	55.3	140.7

Tg: thyroglobulin, Anti-Tg: thyroglobulin antibody, ATA : American Thyroid Association, RAI: radioiodine therapy DT: doubling time (months)

* For patients with positive antibodies, Tg doubling time was not performed #: for biochemical incomplete response, Tumor volume DT was not calculated,

** patient presents with rising antibodies before and after pregnancy N/A: not available.

Regarding the seven patients with biochemical progression, two are undergoing clinical surveillance with laboratory and imaging screening at their last visits. Three of them received radioiodine therapy and progressed with stabilization of serum markers (TG or anti-TG). In two of these patients, the radioiodine treatment was already planned before pregnancy and the disease was considered stable at the last visit. In the third patient, radioiodine treatment was not planned; she presented with disease progression after pregnancy and, at data collection (as per her last visit), had an excellent response after radioiodine. Only two patients (one with indeterminate response and one with biochemical incomplete response) developed biochemical progression that, during the first year after delivery, revealed to be a local metastatic disease (recurrence). On ultrasound screening, these patients presented images suggestive of suspicious lymph nodes that were revealed to be metastatic thyroid carcinoma on fine-needle aspiration biopsy (structural incomplete response). Both these patients remained under active surveillance and had stable disease at data collection.

Of the six patients with structural progression, three had local progression and three had progression of lung metastases. Among the patients with local progression, two had a local metastasis of 1.0 cm that increased to 1.9 cm and 1.8 cm in parallel to increasing non-stimulated TG levels. Both patients underwent neck dissection after pregnancy and had stable disease at data collection. Among the patients with progression of lung metastases, the progression was mild and asymptomatic. At data collection, two of them were under active surveillance and had no indication of systemic therapy, while one had stable disease after radioiodine treatment.

Overall, seven patients received additional therapy within the first 18 months after delivery. In most cases, the additional therapy was already planned before pregnancy due to disease progression, but in two patients, the disease progression occurred after pregnancy and was considered stable at data collection. One of these two patients underwent neck dissection and the other received radioiodine treatment after presenting cervical^131^I uptake on whole-body scanning; both patients were considered to have an excellent response on their last visits.

On univariate analysis, the inclusion in a “high risk of recurrence” category, presence of distant and lymph node metastases at diagnosis, a “structural incomplete response” status before pregnancy, and disease progression before pregnancy emerged as significant factors of disease progression after pregnancy (Table 4). All these factors were confirmed as independent risk factors on multivariate analysis (Table 4). None of the patients died from the disease during follow-up.

**Table 4:** Factors associated with progression

	Progression	Nonprogression	Univariate				Multivariate		
			p-value	HR	CI	p-value	HR	CI	p-value
Age > 30 years	2	15	0.72						
High recurrence risk (ATA 2015)	9	9	**<0.001**	13.5	3.4-53.2	**0.001**	15.8	1.5-163	**0.02**
Tumor size > 4cm	2	7	0.63						
Nodal metastases	11	29	**0.011**	6.44	1.3-31.49	**0.01**	5.3	1.4-25.2	**0.01**
Distant metastasis	4	3	**0.014**	8.8	1.70-46.4	**0.003**	6.55	1.3-30.9	**0.009**
Total Thyroidectomy	12	61	0.435						
RAI	9	47	0.734						
Neck dissection	11	26	0.67						
Structural incomplete response	5	55	**<0.001**	11	2.8-42.0	**0.001**	9.3	1.8-42.0	**0.001**
Progression before pregnancy	6	63	**<0.001**	37.3	12.3-38.8	**<0.001**	40.1	28.9-44.0	**<0.001**

RAI: radioiodine therapy; HR: hazard risk; CI: confidence interval; ATA: American Thyroid Association

### Clinical and disease features of patients diagnosed with DTC before and during pregnancy

The most predominant histological subtype in both groups was papillary thyroid cancer, with a trend toward a greater proportion of the follicular variant of papillary thyroid cancer subtype in the group of women diagnosed during pregnancy (45% versus 18% in the group of women diagnosed before pregnancy). The primary tumor size at diagnosis in both groups was greater than 1 cm, and the median tumor sizes were 2.5 cm in women with DTC diagnosed during pregnancy and 2.2 cm in those with DTC diagnosed before pregnancy. Regarding the presence of local lymph node metastases at diagnosis in women with DTC diagnosed during versus before pregnancy, the rates were 50% and 47%, respectively, for N0, 5% and 18%, respectively, for N1, and 45% and 37%, respectively, for N1b. The corresponding rates of distant metastases at diagnosis were 30% and 9.2%, respectively (p = 0.01).

## DISCUSSION

The results of our study indicate that the impact of pregnancy on DTC appears to be limited. Although this study revealed that disease progression occurred in 17.1% of the cases within the first year after delivery, in most cases, the progression was not clinically significant, and most of the affected patients showed only increased TG levels. Most patients had already presented disease progression before pregnancy, and the pregnancy did not seem to have changed their disease status.

Previous studies have also shown a small impact from pregnancy on the risk of recurrence in women without pre-gestational evidence of biochemical or structural disease, and a slight trend toward progression in women with structural or biochemical incomplete pre-gestational response (7,11,20). Most patients in our study had an excellent response to initial therapy before pregnancy and remained without evidence of biochemical or structural disease during and after pregnancy.

Of note, the patients in our study were young, had well-differentiated disease, and were less likely to have aggressive disease. Although the DTC had a certain impact on pregnancy in some patients, the morbidity was not increased in these patients compared with other young patients treated at our center (15). There were no deaths related to DTC during the entire study.

In a retrospective study of 235 patients, Rakhlin and cols. (11) observed that the response to therapy prior to pregnancy was an excellent indication of progression of structural disease with pregnancy. None of the patients with excellent, indeterminate, or biochemical incomplete responses to therapy prior to pregnancy showed recurrence or progression of structural disease with pregnancy. Only a few patients had TG levels higher after delivery compared with the levels before pregnancy. Among most patients with structural disease, the disease remained stable or progressed slightly. However, a minority (8%) of these patients with structural disease showed an aggressive progression of the lesions detected before pregnancy or developed new disease foci that required additional therapy (surgery or systemic therapy) within 18 months from the delivery. A structural incomplete response before pregnancy had a substantial impact on disease progression after pregnancy (11). We also found that a structural incomplete response and a high initial ATA risk were associated with disease progression after pregnancy. In our cohort, the response to therapy status remained mostly unchanged after pregnancy, similar to the finding by Rakhlin and cols., as did the median TG and anti-TG levels.

According to the 2015 ATA guidelines for the management of thyroid disease during and after pregnancy, monitoring with TG and ultrasound should only be performed during pregnancy in patients with biochemical or structural incomplete response to DTC therapy to identify the few patients with clinically significant risk of structural disease progression. Our data support this approach and prevent excessive ultrasonographic and laboratory tests in patients with excellent and indeterminate response to the initial therapy (22).

Our cohort had 20 patients diagnosed with DTC during pregnancy who underwent thyroidectomy after delivery. The incidence of distant metastases in this group was higher than the rate expected in young patients. This group also had incomplete structural response and disease progression at the last visit. However, the number of patients affected was small and these findings require further confirmation. This observation could be due to the result of hormonal stimulation during pregnancy or selection bias, given that our institution is a referral center for oncology and cares for patients with a more aggressive type of disease, such as those with rapidly growing cervical lesions and symptomatic nodules. We hypothesize that tumors diagnosed during pregnancy could be more responsive to growth stimulation by TSH/β-hCG and were, thus, noticed by palpation during pregnancy. Further studies are required to confirm our findings and determine the potential impact of TSH, β-hCG, and other growth factors on these tumors.

The rate of aggressive disease in our study was greater than the one expected in young women. Indeed, distant metastases were present in 13% of the patients in the entire cohort and in 30% of those diagnosed during pregnancy. This may also be explained by the fact that the INCA is a referral center for oncology in Brazil. In previous studies from our group, we reported that around 35% of our patients with DTC present with AJCC disease stage III or IV and that more than 20% of them show incomplete structural response to initial therapy (15). This could explain a possible bias in sampling patients with more aggressive disease in the present study, resulting in the observation of increased rates of disease progression during pregnancy, which may be a weakness of the present study. Since we showed that the impact of pregnancy in our population was mild, this effect could be even less important in general hospitals, in which the rates of distant metastases are usually as low as 3%-4% (12). This is reassuring, since patients can safely become pregnant even when presenting with evidence of biochemical or structural disease. Another limitation of our study is the small number of patients. However, all patients were followed up at a single center and by the same medical team before, during, and after pregnancy, which probably reduced the bias of medical decisions across different centers. Another fact to consider is the greater effect size in this population, indicating that for a better analysis, a larger number of patients diagnosed during pregnancy is needed. Further studies are needed to better explore those differences.

We excluded patients with cancer diagnosed before the age of 18 years. Pediatric thyroid cancer (23) is known to behave differently depending on the genetic background (24). To avoid this potential bias, we excluded patients with pediatric thyroid cancer, even though that meant excluding a few patients from our analysis. Lastly, we were unable to evaluate the progression rates in patients diagnosed with DTC during pregnancy because surgery was only performed after delivery in all patients.

In conclusion, pregnancy has no substantial impact on the natural course of DTC. Additional therapy in patients with disease progression was already planned before pregnancy. Rates of recurrence and progression after pregnancy were small and perhaps related to a more aggressive disease and a higher initial risk stratification at diagnosis. Still, disease progression may have occurred asymptomatically in some patients. Perhaps in a few patients with this profile, surveillance with imaging is a valid approach at the end of the third trimester of pregnancy and at 2-3 months after delivery. The ATA 2015 initial risk stratification and the dynamic response to therapy pre-pregnancy are good predictors to guide this type of surveillance in pregnancy.
